# Improving patient waiting times and quality of care by arranging access to notes from a neighbouring trust

**DOI:** 10.1192/bjo.2021.478

**Published:** 2021-06-18

**Authors:** Georgios Basdanis, Cormac Fenton

**Affiliations:** Mental Health Liaison Team, St Thomas' Hospital, South London and Maudsley NHS Foundation Trust

## Abstract

**Aims:**

We aim to improve waiting times in the Emergency Department and improve the overall quality of care of out-of-area patients by arranging for the liaison team to have access to the electronic notes system of a neighbouring trust.

**Method:**

St Thomas’ Hospital is located in south London, right opposite the City of Westminster. As a result, approximately 20% of patients we see in mental health liaison are from that locality. Given that they belong to a different trust, we do not have access to their notes, which leads to a delay in trying to establish whether they are known to local mental health services. Often, staff are reluctant to divulge information. When information is shared, it is often late and/or incomplete. We approached the Chief Clinical Information Officer and Head of Information Governance from Central and North West London (CNWL) NHS Foundation Trust. We held weekly meetings which included both IT departments. Our IT had to install the electronic notes application (SystmOne) on our computers and open relevant firewall ports. The information is access through an NHS Smartcard, therefore CNWL had to authorise read-only Smartcard profiles for every member of the liaison team. A quick reference guide was created for all staff that would be using the new application. The system went live on 21 January 2021.

**Result:**

We audited patient outcomes in December 2020 and February 2021 for initial comparison. In December 2020, the median time from referral to discharge was 6 hours 35 minutes. 25% of patients were admitted and 17% discharged with HTT. In February 2021, the median time from referral to discharge was 3 hours 19 minutes. 16% of patients were admitted and 5% discharged with HTT.

**Conclusion:**

It is likely that by reducing the time required for collateral information, overall waiting times in the emergency department will be reduced. Clinicians are likely to feel more confident in their discharge planning if they have access to all clinical notes and previous risk assessments, which might in turn reduce referrals to HTT or admission. There should be further attempts by neighbouring NHS trusts, especially in London, to ensure access to their electronic notes system in order to reduce waiting times and improve the quality of patient care. We have already been approached for more information by a trust in North London who are interested in establishing access to a neighbouring trust's notes.

